# Modelling far field pacing for terminating spiral waves pinned to ischaemic heterogeneities in cardiac tissue

**DOI:** 10.1098/rsta.2016.0289

**Published:** 2017-05-15

**Authors:** E. Boccia, S. Luther, U. Parlitz

**Affiliations:** 1 Max Planck Institute for Dynamics and Self-Organization, Am Fassberg 17, 37077 Göttingen, Germany; 2 Institute for Nonlinear Dynamics, Georg-August-Universität Göttingen, Friedrich-Hund-Platz 1, 37077 Göttingen, Germany; 3 Institute of Pharmacology and Toxicology, University Medical Center, Robert-Koch-Strasse 40, 37075 Göttingen, Germany; 4 Department of Bioengineering, Northeastern University, 360 Huntington Avenue, Boston MA 02115, USA; 5 Department of Physics, Northeastern University, 360 Huntington Avenue, Boston MA 02115, USA

**Keywords:** excitable media, anisotropy, cardiac dynamics, virtual electrodes, vulnerable window, defibrillation

## Abstract

In cardiac tissue, electrical spiral waves pinned to a heterogeneity can be unpinned (and eventually terminated) using electric far field pulses and recruiting the heterogeneity as a virtual electrode. While for isotropic media the process of unpinning is much better understood, the case of an anisotropic substrate with different conductivities in different directions still needs intensive investigation. To study the impact of anisotropy on the unpinning process, we present numerical simulations based on the bidomain formulation of the phase I of the Luo and Rudy action potential model modified due to the occurrence of acute myocardial ischaemia. Simulating a rotating spiral wave pinned to an ischaemic heterogeneity, we compare the success of sequences of far field pulses in the isotropic and the anisotropic case for spirals still in transient or in steady rotation states. Our results clearly indicate that the range of pacing parameters resulting in successful termination of pinned spiral waves is larger in anisotropic tissue than in an isotropic medium.

This article is part of the themed issue ‘Mathematical methods in medicine: neuroscience, cardiology and pathology’.

## 1. Introduction

Cardiac tissue is a nonlinear excitable medium whose spatio-temporal dynamics exhibits a complex behaviour ranging from regular to turbulent activity. The first is associated with plane waves, generated by specific pacemaker cells and travelling through the tissue triggering a coordinated mechanical contraction of the myocardium. Turbulent activity, instead, arises due to dysfunctions affecting the propagation of the electric pulse and seriously affects the pumping function of the heart, leading to the occurrence of life-threatening diseases, i.e. cardiac arrhythmias. Asynchronous electromechanical activity in the heart is known in the literature as fibrillation. During fibrillation, the regular heart contraction is altered by fast re-entrant waves (spiral or scroll waves) [1–4], which find a very critical substrate in the heterogeneous and anisotropic structure of the cardiac tissue [5]. Spiral and scroll waves can in fact get anchored (pinned) to localized heterogeneities, giving rise to abnormal high-frequency heart rhythms leading to the aforementioned arrhythmias. For this reason, if not followed by repinning, unpinning is an important step towards successful termination of re-entries.

In this scenario, one of the most challenging goals is controlling the complex dynamics of the heart under conditions of fibrillation. In clinical practice, the most often used and effective approach against re-entrant activity in the heart consists of delivering a high-energy electric shock that excites the entire medium at once and sets all the cells to their refractory state, thus terminating any electrical activity. Although this *defibrillation* technique is used routinely, the delivery of high-energy pulses is associated with undesired side effects that seriously affect the successful outcome of the therapy [6–8].

For this reason, there is a strong need for low-energy control methods. The approach presented in this paper is based on far field pacing (FFP), which recruits both the intrinsic (due to the complex geometry) and the induced (due to pathological conditions) multi-sized heterogeneities of the heart tissue to successfully terminate arrhythmias [9]. A heterogeneity represents an area of conduction changes, and when an electric field is applied to the whole heart, areas of depolarization and hyperpolarization (so-called Weidmann zones) [10] appear at the boundary of this ‘obstacle’ due to a redistribution of intracellular and extracellular currents. If the depolarization exceeds the excitation threshold, the heterogeneity acts as a source of excitation, i.e. a *virtual electrode* [11–17]. The intensity of the external electric field applied in FFP is much weaker than the defibrillation threshold [18]. It has been demonstrated both numerically and experimentally that waves emitted by virtual electrodes can be used to unpin or terminate re-entrant waves pinned to heterogeneities [19–21].

Detailed studies have been conducted in order to better understand these mechanisms and their impact on the success rate of FFP applied to isotropic media [12,19,22]. These investigations introduced concepts such as vulnerability and vulnerable window [19,23,24] and their relationship with the unpinning window, and refractory and excitable periods [19] on the obstacle’s boundary to which the spiral is pinned.

Intensive investigation is still needed to translate these mechanisms in anisotropic media, where the role played by fibres and different conductivities in different directions has been demonstrated to be fundamental in order to obtain reliable simulation results [25,26]. This paper focuses on the effects exerted by anisotropy on the process of unpinning and termination of pinned spirals. We implement a bidomain formulation of phase I of the Luo and Rudy action potential model under conditions of acute ischaemia. The bidomain model is a continuum model in which the microstructure of the myocardium is replaced by intracellular and extracellular domains, separated by the cellular membrane. Therefore, it is the most realistic mathematical expression for macroscopic simulation of cardiac tissue [27]. The two spaces have different electrical conductivities and in each domain anisotropy is reproduced by assigning different conductivity values in the directions parallel and perpendicular to myocardial fibres. Termination of a spiral pinned to an ischaemic heterogeneity is attempted through the delivery of sequences of pulses in FFP at different pacing frequencies and at different angles of the pinned spiral rotating around the heterogeneity. The paper is organized as follows: §2 introduces the modelling used in this article; §§3 and 4 report the results of FFP applied in an isotropic and in an anisotropic medium, respectively; §5 sums up and compares the outcomes of the two previous sets of simulations.

## 2. Simulations

The impact of anisotropy on wave dynamics is investigated by means of a bidomain model of cardiac tissue [27].

The transmembrane potential *V*_m_ and the extracellular potential *V*_e_ are governed by
2.1
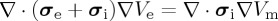
and
2.2
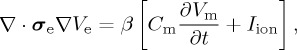
where *β* is the ratio of the membrane surface area to the tissue volume, *C*_m_ is the membrane capacitance per unit area, ***σ***_e_ and ***σ***_i_ are the diagonal extracellular and intracellular conductivity tensors, respectively (each one resolved into two components: one parallel (*σ*_*L*_) and one perpendicular (*σ*_*T*_) to the fibres), and *I*_ion_ is the sum of the transmembrane ionic currents described by the local ionic model, being in our sets of simulations phase I of the Luo and Rudy action potential model [28].

Simulations are run in a two-dimensional sheet of cardiac tissue (4 cm × 4 cm) with a circular ischaemic heterogeneity (radius 0.6 cm) in the middle of it. In order to simulate acute ischaemia, the ionic model is modified accordingly. The ischaemic area is treated as a region with reduced conduction properties where conditions of hyperkalaemia and acidosis arising at the cellular level are modelled. The extracellular potassium concentration, [*K*^+^]_*o*_, is incremented from 5.4 to 14 mmol l^−1^ [29] due to hyperkalaemia; the sodium and L-type calcium channel-specific conductances, *g*_Na_ and *g*_Ca_, respectively (both chosen according to Luo & Rudy [28]), are both reduced by 25% [30] as an effect of acidosis. Changes in intracellular and extracellular conductivities are simulated according to the experimental data provided by Kléber & Riegger [31]. After the arrest of coronary flow, extracellular resistance is supposed to increase immediately by approximately 30%, due to a drop in perfusion pressure and a diminution of intravascular volume. After approximately 20 min, a second gradual increase up to 80% occurs. Regarding the intracellular resistance, it is supposed to remain constant in the first 10–12 min, followed by a steep increase due to the progression of cellular uncoupling and the breakdown of cellular homeostasis. Considering these phenomena and assuming that the dynamics of the system is simulated approximately 20 min after the onset of ischaemia, bidomain conductivities in the ischaemic heterogeneity are reduced by 40% in the extracellular domain and by 50% in the intracellular domain [31].

Each pulse is delivered for 5 ms in FFP by plane electrodes placed perpendicular to the longitudinal direction (*x*-axis = direction of the applied electric field). During the pulses, the boundary conditions are imposed according to Fishler [15]: for a longitudinally oriented shock, in both the intracellular and the extracellular domains potentials on the transverse boundary are governed by Neumann no-flux boundary conditions, while potentials along the longitudinal boundaries are governed by Dirichlet boundary conditions. In particular for Dirichlet boundary conditions,
2.3
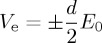
and
2.4
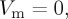
where *d* is the edge length of the two-dimensional simulated tissue. Equation (2.3) ensures the application of a uniform electric field with intensity *E*_0_ and direction parallel to the fibres. Using condition (2.4) avoids the near-field effects on *V*_m_ at the tissue edge and thus allows the focus to be only on the impact of far field stimulations. During intervals between pulses, instead, Neumann no-flux conditions are applied at all boundaries.

The set of parameters adopted in both the isotropic and the anisotropic computations are reported in table 1.

**Table 1.** Set of parameters adopted for the simulations in both the isotropic and anisotropic domains. All the values refer to healthy conditions; changes due to ischaemia are described in the text.

	isotropic tissue	anisotropic tissue
*β* (cm^−1^)	0.2	0.2
*C*_m_ (μF cm^−2^)	1	1
*g*_Na_ (mS^−1^ cm^2^)	23	23
*g*_Ca_ (mS^−1^ cm^2^)	0.09	0.09
[*K*^+^]_*o*_ (mmol^−1^ l)	5.4	5.4
*σ*_e*T*_ (mS cm^−1^)	4.20	2.36
*σ*_e*L*_ (mS cm^−1^)	4.20	6.25
*σ*_i*T*_ (mS cm^−1^)	0.19	0.19
*σ*_i*L*_ (mS cm^−1^)	0.19	1.74
*E*_0_ (V cm^−1^)	45	9

## 3. Isotropic medium

In this section, we present one simplifying assumption often used in bidomain models of cardiac tissue: the ‘isotropic bidomain’ approximation [32]. It affects the transversal and longitudinal conductivities that are set to be the same in both the intracellular and extracellular spaces, i.e. *σ*_i*T*_=*σ*_*iL*_=*σ*_i_ and *σ*_e*T*_=*σ*_*eL*_=*σ*_e_. This assumption removes the presence of fibres from the model of the tissue.

Assigning conductivity values in bidomain model simulations is not straightforward: although these parameters have been measured experimentally [33–37], the data are inconsistent and therefore no consensus exists regarding their accurate values. In both isotropic and anisotropic media, our simulations refer to the experimental values provided by Clerc [33].

**Figure 1.**
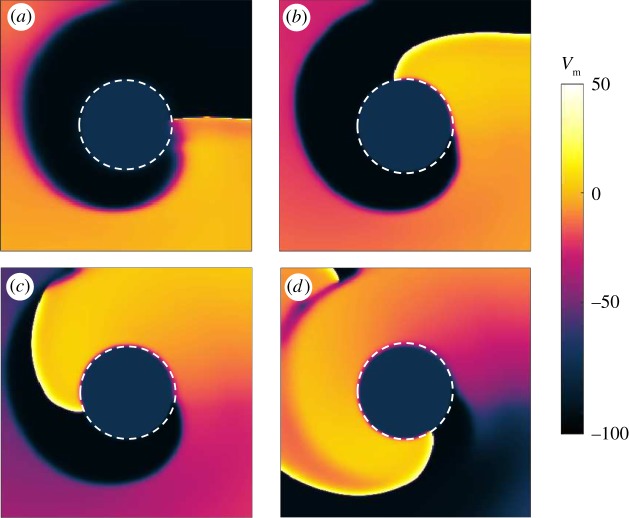
Snapshots showing the rotation of a spiral pinned to the ischaemic heterogeneity (radius 0.6 cm, highlighted by the white dashed circle) in an isotropic medium (4 cm×4 cm). Without external stimulation the spiral is stably pinned to the obstacle and a rotation period (*T*_sp_) of 380 ms is recorded. Positions of the spiral tip on the border of the ischaemic area are shown at 0 ms (*a*), 100 ms (*b*), 200 ms (*c*) and 340 ms (*d*) within one rotation period. (Online version in colour.)

Figure 1 shows snapshots taken from a simulation computing the rotation of a spiral pinned to the ischaemic heterogeneity (highlighted by the white dashed circle). In the absence of external stimulation, the spiral is stably pinned to the obstacle and a rotation period (*T*_sp_) of 380 ms is recorded. The positions of the spiral tip on the border of the ischaemic area are shown at 0 ms (figure 1*a*), 100 ms (figure 1*b*), 200 ms (figure 1*c*) and 340 ms (figure 1*d*), within one rotation period. The panels in figure 1 show the states of the spiral taken during an early phase of the pinning process, in which the spiral shows a stable behaviour in terms of constant speed and persistence of pinning in the absence of external perturbation, but has not yet spent a ‘sufficiently long time’ in this condition to be in its full steady rotation. The motivation behind the choice of a transient behaviour will be clarified further in the text.

For the stimulation protocol, different times for delivering the first pulse (*t*_1_) were chosen, corresponding to different angles of the spiral tip on the boundary of the ischaemic zone. This means that the positions of the spiral tip were chosen at an interval of 20 ms within one rotation period and each of them was set as the initial condition for a set of simulations. Figure 1 provides four examples of initial conditions at which the first pulse was delivered. In each set of simulations, the interval between pulses (*T*_p_) was set taking into account two approaches of electrical stimulation: *underdrive*^1^ and *overdrive*^2^ pacing. Therefore, according to the first method, *T*_p_=1.1*T*_sp_, *T*_p_=1.3*T*_sp_, *T*_p_=1.5*T*_sp_, *T*_p_=1.7*T*_sp_, *T*_p_=1.9*T*_sp_. For overdrive pacing, instead, *T*_p_=0.1*T*_sp_, *T*_p_=0.3*T*_sp_, *T*_p_=0.5*T*_sp_, *T*_p_=0.7*T*_sp_, *T*_p_=0.9*T*_sp_. The number of delivered pulses ranged from one to five and the outcome of a stimulation (maintenance, unpinning or termination of the pinned spiral) was checked at each interval immediately before the potential application of another pulse. At this point, the pacing protocol was considered to be successful and thus interrupted only in the case of spiral termination.

The intensity of the electric field, *E*_0_, represents the minimum intensity which, under isotropic conditions, induces a depolarization that exceeds the excitation threshold at the boundary of the obstacle and creates a propagating wave.

**Figure 2.**
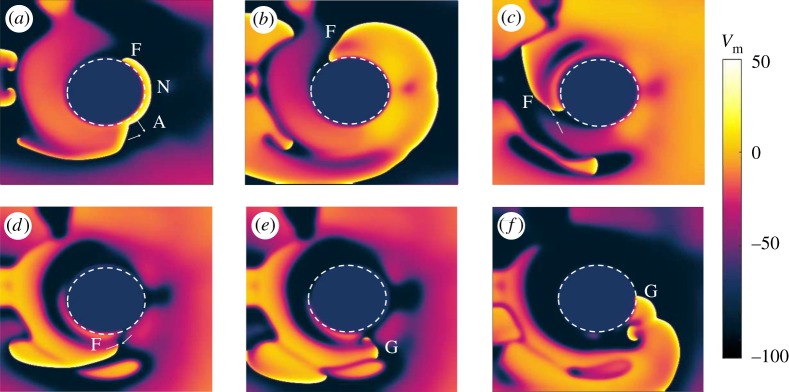
Isotropic medium: example of unpinning and repinning of the pinned spiral during overdrive pacing (*T*_p_=0.9*T*_sp_) with *t*_1_=360 ms, after the delivery of the third pulse. In (*a*), the application of the electric field induces areas of depolarization and hyperpolarization at the boundaries of the ischaemic zone. Since the excitation threshold is exceeded, a new wave (N) is generated. One of the ends of the newly initiated wave (A) collides with the original spiral and annihilates it. The other end (F) travels counterclockwise around the boundary of the obstacle (*b*), but its propagation is inhibited by the refractory tail of the original spiral wave (*c*) and (*d*). This causes the temporary detachment of the wave (G) (*e*), followed by reattachment, also called repinning (*f*). The snapshots are taken at 15 ms (*a*), 70 ms (*b*), 170 ms (*c*), 270 ms (*d*), 300 ms (*e*) and 385 ms (*f*) after the delivery of the pulse. (Online version in colour.)

**Figure 3.**
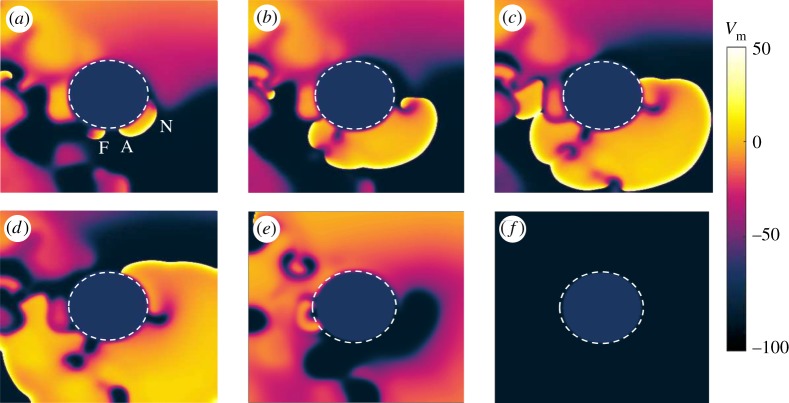
Isotropic medium: example of successful termination of the pinned spiral. The simulation is run in underdrive pacing (*T*_p_=1.9*T*_sp_) for *t*_1_=80 ms and snapshots are taken from the interval following the delivery of the third stimulus. In (*a*) a new wave N is induced at the obstacle by the pulse, but this time part of the boundary is still refractory and does not get excited by the new shock. Therefore, only one end of the wave (A) is able to propagate and to collide with the surrounding waves (in particular with the small active pinned wave F) (*a*). The dynamics of the system then undergoes a transient (*c*–*e*), showing wave annihilation and break-up. At the end, the transient results in a stable regime in which the electrical activity in the tissue has been completely terminated (*f*). The snapshots are taken at 25 ms (*a*), 75 ms (*b*), 120 ms (*c*), 185 ms (*d*), 350 ms (*e*) and 700 ms (*f*) after the delivery of the pulse. (Online version in colour.)

Figures 2 and 3 show two interesting phenomena arising in an isotropic tissue during FFP stimulation: unpinning and termination of a pinned spiral, respectively.

Figure 2 shows snapshots taken from a simulation implementing overdrive pacing (*T*_p_=0.9*T*_sp_) with *t*_1_=360 ms, after the delivery of the third pulse. In figure 2*a*, the application of the electric field induces areas of depolarization and hyperpolarization at the boundaries of the ischaemic zone. Since the excitation threshold is exceeded, a new wave (N) is generated. One of the ends of the newly initiated wave (A) collides with the original spiral and annihilates it. The other end (F) travels counterclockwise around the boundary of the obstacle (figure 2*b*), but its propagation is inhibited by the refractory tail of the original spiral wave (figure 2*c*,*d*). This causes the temporary detachment of the wave (G) (figure 2*e*), followed by reattachment, also called repinning (figure 2*f*). The snapshots are taken at 15 ms (figure 2*a*), 70 ms (figure 2*b*), 170 ms (figure 2*c*), 270 ms (figure 2*d*), 300 ms (figure 2*e*) and 385 ms (figure 2*f*) after the delivery of the pulse.

Figure 3 shows an example of successful termination of the pinned spiral. The simulation is run in underdrive pacing (*T*_p_=1.9*T*_sp_) for *t*_1_=80 ms and snapshots are taken from the interval following the delivery of the third stimulus. In figure 3*a*, a new wave N is induced at the obstacle by the pulse, but this time part of the boundary is still refractory and does not get excited by the new pulse. Therefore, only one end of the wave (A) is able to propagate and to collide with the surrounding waves (in particular with the small active pinned wave F; figure 3*a*). The dynamics of the system then undergoes a transient (figure 3*c*–*e*), showing wave annihilation and break-up. At the end, the transient results in a stable regime in which the electrical activity in the tissue has been completely terminated and figure 3*f* shows the resting state of the simulated domain. The snapshots are taken at 25 ms (figure 3*a*), 75 ms (figure 3*b*), 120 ms (figure 3*c*), 185 ms (figure 3*d*), 350 ms (figure 3*e*) and 700 ms (figure 3*f*) after the delivery of the pulse.

**Figure 4.**
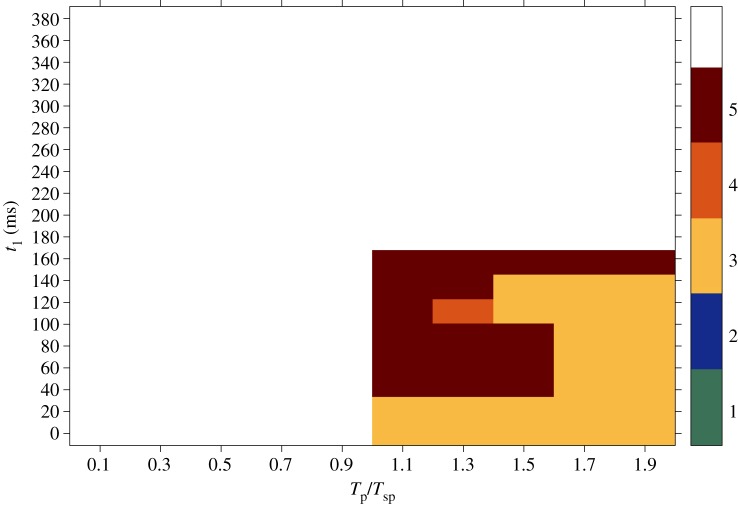
Results obtained from simulations in the isotropic case. On the *x*-axis different time intervals between pulses (*T*_p_) relative to the period of the spiral (*T*_sp_) are shown, varying from overdrive to underdrive pacing. The *y*-axis reports several initial conditions at which the first pulse was delivered (*t*_1_), each one corresponding to a specific position of the spiral on the border of the heterogeneity within one rotation period (cf. figure 1). The outcome is expressed in terms of the number of pulses that have to be applied in order to terminate the pinned spiral (as shown in figure 3*f*). The maximum number of stimulations delivered during the pacing protocol was five, so the white area in the graph corresponds to sequences that were not successful within the five pulses. For this reason, no statement can be made about the possibility of terminating the spiral wave in that region. (Online version in colour.)

The results obtained from simulations in the isotropic case are summarized in figure 4. The graph shows the success rate for sequences of far field pulses delivered at a certain interval between pulses (on the *x*-axis, varying from overdrive to underdrive pacing) and starting from a certain initial condition (on the *y*-axis, where each value corresponds to a specific position of the spiral on the border of the heterogeneity within one rotation period; cf. figure 1). The outcome is expressed in terms of the number of pulses that have to be applied in order to terminate the pinned spiral (as shown in figure 3*f*). As already mentioned, the maximum number of stimulations delivered during the pacing protocol was five, so the white area in the graph corresponds to sequences that were not successful within the five pulses. For this reason, no statement can be made regarding the number of pulses required for that region.

Regarding successful pulses, it is worth noting that at least three shocks have to be applied in order to terminate the pinned spiral. Moreover, the area of the graph where successful pulse sequences take place is restricted to the ‘underdrive region’ [22,38].

## 4. Anisotropic medium

In this section, we present the results for unpinning and termination of a pinned spiral in an anisotropic medium. In this case no simplifying assumption was made for the bidomain model, but four different conductivities were introduced, as shown in table 1. Myocardial fibres are modelled as straight, uniform and directed along the *x*-direction.

**Figure 5.**
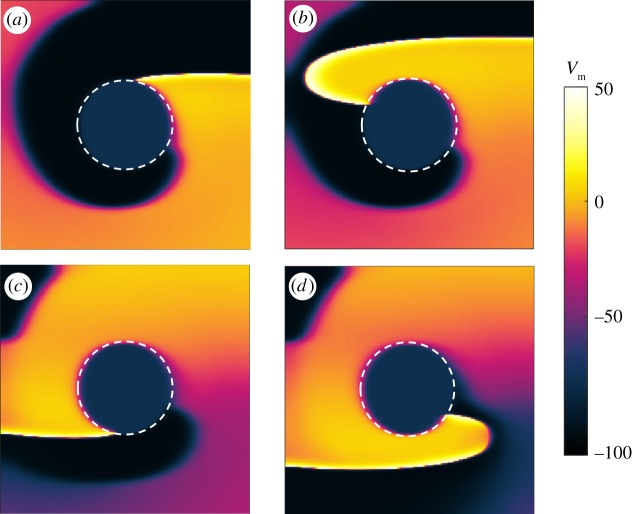
Snapshots showing the rotation of a spiral pinned to the ischaemic heterogeneity (radius 0.6 cm, highlighted by the white dashed circle) in an anisotropic medium (4 cm × 4 cm). Without external stimulation the spiral is stably pinned to the obstacle and a rotation period (*T*_sp_) of 350 ms is recorded. The positions of the spiral tip on the border of the ischaemic area are shown at 80 ms (*a*), 140 ms (*b*), 250 ms (*c*) and 300 ms (*d*) within one rotation period. (Online version in colour.)

Figure 5 shows snapshots taken from a simulation implementing the rotation of the spiral pinned to the ischaemic heterogeneity without any far field stimulations. Under these conditions, in which anisotropy ratios in the intracellular and extracellular domains are approximately 9 and 2.6 [33], respectively, the rotation period (*T*_sp_) of the spiral changes to 350 ms. The positions of the spiral tip on the boundary of the ischaemic area are recorded at 80 ms (figure 5*a*), 140 ms (figure 5*b*), 250 ms (figure 5*c*) and 300 ms (figure 5*d*) within one rotation period.

As already mentioned for the isotropic domain, these snapshots refer to the spiral during its transient behaviour. The spiral has a constant period and a ‘sufficiently strong’ pinning to the heterogeneity, requiring external perturbations in order to be detached from it.

The pacing protocol adopted in this section was the same as the one performed for isotropic computations and described in §3. Each set of simulations had initial conditions chosen at different positions of the spiral tip on the heterogeneity, which correspond to the time at which the first pulse was delivered (*t*_1_). Again, the difference between the *t*_1_ values was set to 20 ms, in order to cover a wide range of spiral tip positions in the application of the first stimulation. The interval between pulses ranged from overdrive to underdrive pacing.

The intensity of the electric field *E*_0_ is different from the isotropic simulations, since this time a smaller value was already sufficient to induce a propagating wave out of the obstacle.

In contrast with the results shown in §3, anisotropic simulations resulted either in the maintenance or in the termination of the spiral, but no episodes of unpinning were observed.

**Figure 6.**
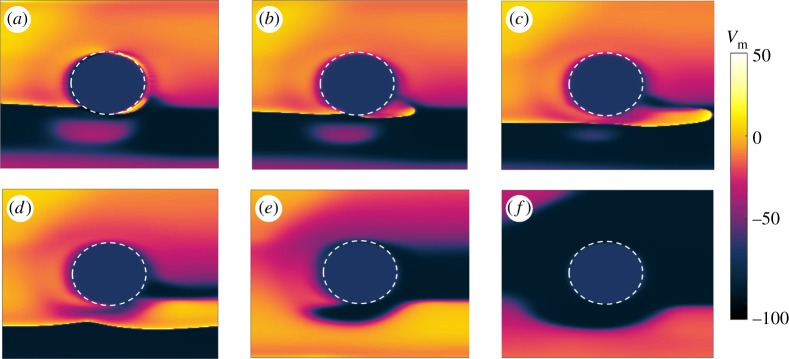
Anisotropic medium: example of successful termination of the pinned spiral after the second stimulus has been delivered. The overdrive pacing protocol was applied, with (*T*_p_=0.9*T*_sp_) and *t*_1_=300 ms. In (*a*) the applied far field pulse is strong enough to exceed the excitation threshold and to induce a new wave nucleating from the heterogeneity. Since part of the boundary is still refractory due to the tail of the original spiral, only a small portion of the new wave is able to propagate. Within this part, one end disconnects from the obstacle because its propagation is inhibited by the refractory tail of the spiral, as shown in (*b*). The other end forms a pinned wave and collides with the tip of the original spiral (*c*,*d*). Panel (*e*) shows how this collision leads to the annihilation of the two waves and hence to the termination of the pinned spiral, as shown in figure 6*f*. The snapshots are taken at 3 ms (*a*), 14 ms (*b*), 34 ms (*c*), 64 ms (*d*), 134 ms (*e*) and 180 ms (*f*) after the delivery of the pulse. (Online version in colour.)

Snapshots from a simulation resulting in successful termination of the pinned spiral after the second stimulus are shown in figure 6. The overdrive pacing protocol was applied, with (*T*_p_=0.9*T*_sp_) and *t*_1_=300 ms. In figure 6*a*, the applied far field pulse is strong enough to exceed the excitation threshold and to induce a new wave nucleating from the heterogeneity. Since part of the boundary is still refractory due to the tail of the original spiral, only a small portion of the new wave is able to propagate. Within this part, one end disconnects from the obstacle because its propagation is inhibited by the refractory tail of the spiral, as shown in figure 6*b*. The other end forms a pinned wave and collides with the tip of the original spiral (figure 6*c*,*d*). Figure 6*e* shows how this collision leads to the annihilation of the two waves and hence to the termination of the pinned spiral, as shown in figure 6*f*. The snapshots are taken at 3 ms (figure 6*a*), 14 ms (figure 6*b*), 34 ms (figure 6*c*), 64 ms (figure 6*d*), 134 ms (figure 6*e*) and 180 ms (figure 6*f*) after the delivery of the pulse.

**Figure 7.**
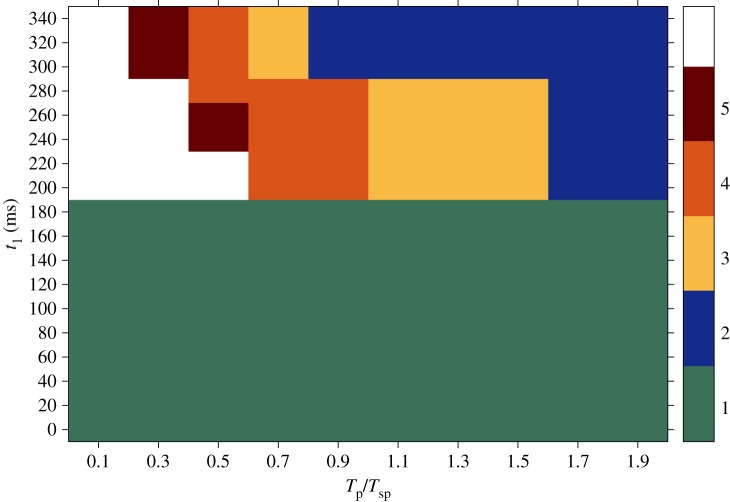
Results obtained from simulations in the anisotropic case during the transient regime. On the *x*-axis different time intervals between pulses (*T*_p_) relative to the period of the spiral (*T*_sp_) are shown, varying from overdrive to underdrive pacing. The *y*-axis reports several initial conditions at which the first pulse was delivered (*t*_1_), each one corresponding to a specific position of the spiral on the border of the heterogeneity within one rotation period; compare with figure 5. The outcome is expressed in terms of the number of pulses that have to be applied in order to terminate the pinned spiral (as shown in figure 6*f*). The maximum number of stimulations delivered during the pacing protocol was five, so the white area in the graph corresponds to sequences that were not successful within the five pulses. For this reason, no statement can be made about the possibility of terminating the spiral wave in that region. (Online version in colour.)

An overview of the results obtained from the anisotropic simulations with respect to the time interval between pulses and the time of the first pulse *t*_1_ within one spiral rotation is shown in figure 7. The plot reports the success rate of the pacing protocols in terms of the number of pulses (ranging from 1 to 5) that it is necessary to deliver in order to achieve the termination of the pinned spiral. As in the previous case, on the *x*-axis the time interval spans both the overdrive and the underdrive pacing protocols; on the *y*-axis a set of 18 initial conditions covers a wide range of positions taken by the pinned spiral tip during one rotation (cf. figure 5).

In this case, successful termination is already obtained after the delivery of only one pulse for most positions of the spiral tip on the boundary of the obstacle. In addition, most of the combinations of parameters result in spiral termination within the delivery of five pulses (the white region, in which no statement can be made, is much smaller than that in figure 4). The most successful episodes in the upper graph, i.e. terminations requiring a smaller number of pulses, tend to gather in the underdrive region.

All the results shown so far for the anisotropic case refer to the transient behaviour of the pinned spiral. Further computations have been carried out in order to investigate the impact that the different (transient or asymptotic) states of the spiral dynamics can have on the outcome of the pulses. For this reason, the same pacing protocol previously described was also applied during the asymptotic regime of the spiral and the success rate was computed.

**Figure 8.**
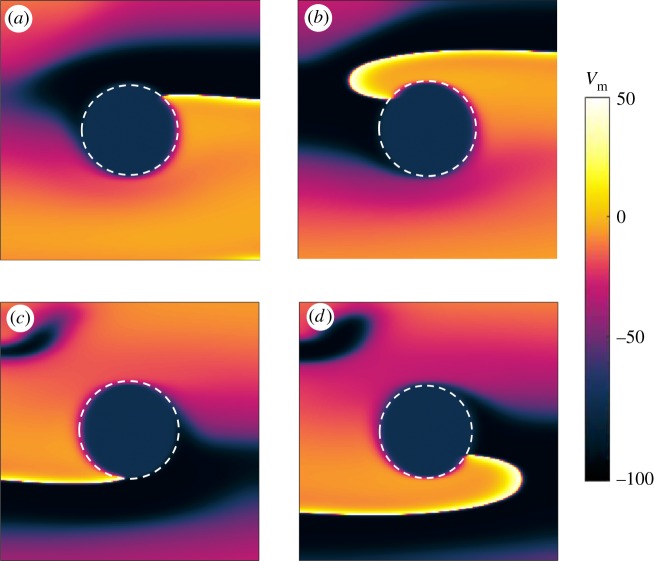
Snapshots showing the rotation of a spiral pinned to the ischaemic heterogeneity (radius 0.6 cm, highlighted by the white dashed circle) in an anisotropic medium (4 cm×4 cm) during the asymptotic regime. Without external stimulation the spiral is stably pinned to the obstacle and a rotation period (*T*_sp_) of 340 ms is recorded. Positions of the spiral tip on the border of the ischaemic area are shown at 40 ms (*a*), 110 ms (*b*), 220 ms (*c*) and 280 ms (*d*) within one rotation period. (Online version in colour.)

Figure 8 shows snapshots of the spiral pinned to the ischaemic heterogeneity without any far field stimulations during steady rotation. The period of the spiral in this new dynamic state changed from 350 ms to 340 ms. Snapshots show the positions of the spiral tip on the boundary of the ischaemic area recorded at 40 ms (figure 8*a*), 110 ms (figure 8*b*), 220 ms (figure 8*c*) and 280 ms (figure 8*d*) within one rotation period.

**Figure 9.**
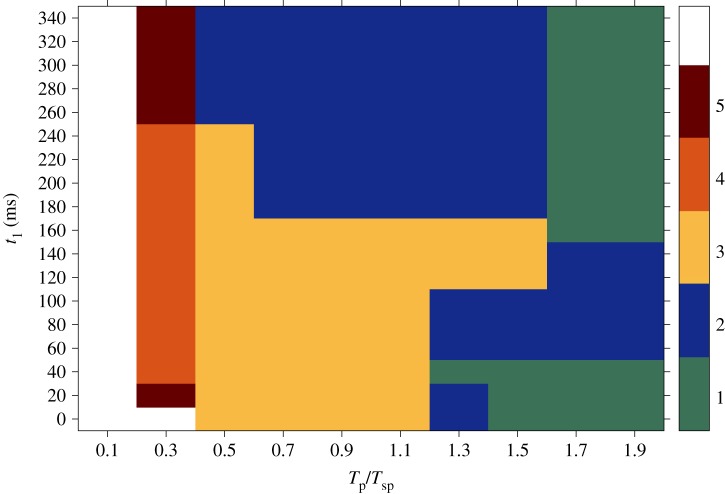
Results obtained from simulations in the anisotropic case during an asymptotic regime. On the *x*-axis different time intervals between pulses (*T*_p_) relative to the period of the spiral (*T*_sp_) are shown, varying from overdrive to underdrive pacing. The *y*-axis reports several initial conditions at which the first pulse was delivered (*t*_1_), each one corresponding to a specific position of the spiral on the border of the heterogeneity within one rotation period (cf. figure 8). The maximum number of stimulations delivered during the pacing protocol was five, so the white area in the graph corresponds to sequences that were not successful within the five pulses. For this reason, no statement can be made about the possibility of terminating the spiral wave in that region. (Online version in colour.)

The success rate of the protocol in the new dynamic state is shown in figure 9. As in figures 4 and 7, the results summarized in figure 9 report the number of pulses (ranging from one to a maximum of five) that enable termination of the spiral. The *x*-axis is partitioned into overdrive and underdrive regions (depending on the values of the ratio *T*_p_/*T*_sp_); the *y*-axis reports the values of *t*_1_ (as previously defined), of which some examples are shown in figure 8. Similar to the transient regime shown in figure 7, there is a green area in figure 9 where the first pulse is already sufficient to terminate the pinned spiral. The main difference consists in the extension of this area, which in figure 7 spreads from the underdrive to the overdrive region and covers a wide range of parameter combinations, while in figure 9 it ‘shrinks’ to a much smaller area in the underdrive region. Note that, in both figure 7 and figure 9, the most successful cases occur for underdrive pacing. In addition, figure 9 shows that in most cases five pulses (or fewer) are sufficient to terminate a steadily rotating spiral.

## 5. Conclusion

In this paper, we demonstrate the crucial role of anisotropy in the process of termination of a spiral pinned to an ischaemic heterogeneity during far field stimulation. A comparison between results obtained under isotropic and anisotropic conditions highlights the fundamental differences that must be taken into account when comparing simulations with experimental results. Simplifying assumptions in the conductivities of the bidomain model (as the ‘isotropic bidomain’ approximation) drastically reduce the computational load of simulations, but have been demonstrated both analytically and numerically to be in disagreement with experimental observations [26]. Therefore, unequal anisotropy ratios must be used in order to achieve reliable results [26,39].

Furthermore, we compared the impact of the external field pulse on spirals at an early stage when they were still in the transient regime (figures 4 and 7) with spirals that had already reached the asymptotic state of steady rotations (figure 9). This comparison was motivated by clinical practice, when it is important to have rapid and immediate intervention, because waiting too long before defibrillating the heart can result in the death of the patient. Therefore, with the aim of investigating the critical role played by the ‘waiting time’ and the dynamic state of the heart tissue during defibrillation, figure 9 has been computed to measure the success rate of defibrillation attempts during steady rotation of the pinned spiral.

In our simulations, the main differences between isotropic and anisotropic simulations lie in the amount of energy required to recruit the heterogeneity as a virtual electrode and in the probability of successful termination assigned to a specific sequence of pulses.

In more detail, the intensity of the electric field applied at the virtual electrodes required to exceed the excitation threshold and induce a propagating wave at the boundary of the ischaemic area was five times stronger in the isotropic case than in the one used in the anisotropic case due to different conductivity values. Interestingly, we found that, although a higher amount of energy was released in the isotropic regime, the probability of successful termination was lower. This statement is supported by the comparison between figures 4, 7 and 9.

During the transient regime, in the isotropic case, a higher number of pulses have to be delivered in order to induce termination of the pinned spiral. As shown in figure 4, a sequence of pulses starts to be successful only after the delivery of the third stimulation, while the outcome from anisotropic simulations shows a region where the first pulse is already sufficient to achieve the goal (figure 7). A smaller intensity of the electric field in anisotropic tissues also ensures spiral termination within the five pulses for a larger range of parameter combinations, as shown by the smaller white area in figure 7. Checking the outcome of a stimulation (maintenance, unpinning or termination of the pinned spiral) at each interval immediately before the potential application of another pulse allowed the protocol to be stopped as soon as termination was detected. In this way, it was possible to avoid the delivery of unnecessary pulses. In this last case, one would expect that, in the case of completely quiescent tissue, the delivery of a new pulse and the recruitment of the heterogeneity as a virtual electrode should not alter the success rate, since the new wave would spread in the tissue without hitting any further obstacles. The results could be very different in the case of existing residual waves propagating in the tissue at the moment when a new far field pulse is applied and a new wave originates from the heterogeneity. In these circumstances, the collision between the waves might lead to a more complex dynamics that might affect the success rate of the defibrillation attempts. When translating this pacing protocol to clinical practice (in experiments or in modern pacemakers, for example), one may think of regulating the electrical behaviour of the heart by sensing and stimulating activities using specific electrodes implanted at different points in the heart. When abnormal activity due to incoming arrhythmias is detected by the sensing electrodes, a variable number of external pulses could be delivered by the stimulating electrodes until the physiological sinus rhythm is restored and the defibrillation attempt is considered successful.

The results from our simulations show that successful termination in anisotropic media is not restricted to underdrive pacing, as in the case of isotropic substrates [22], but it also extends to overdrive pacing. The exact mechanism that underlies this behaviour in the anisotropic computations still has to be completely understood. A preliminary guess could be made by comparing figures 7 and 9, i.e. transient and steady rotation of the pinned spiral. Our results show that the transient regime seems somehow to support the process of spiral termination, since the spiral dynamics is less stable than in its steady state and, for this reason, perturbations by external far field pulses are more likely to result in spiral termination. This ‘vulnerability’ of the spiral, together with the higher conductivity values in the longitudinal direction, i.e. the direction of the fibres, might induce a major drift of the spirals towards the boundary of the tissue and cause their annihilation. However, as previously stated, this interpretation is only preliminary and would require additional simulations to explore a broader range of possible scenarios.

Another interesting observation that we made was the occurrence of unpinning and repinning of the spiral only in isotropic simulations. These phenomena have been observed in previous isotropic investigations, where the concepts of unpinning and vulnerable window have been studied [18,19]. Further research is needed to properly translate the mechanisms of re-entrant dynamics into anisotropic computations. This includes the impact of the relative angle between the direction of the electric field and the orientation of the myocardial fibres. In this study, as a first approach, an electric field parallel to myocardial fibres has been modelled, but we expect the relative angle to be a ‘critical parameter’. To summarize, our results provide clear evidence of the strong impact exerted by anisotropy in the tissue and of misleading results that can be obtained when introducing any simplifying approximations for the conductivity values in bidomain modelling.

## Footnotes

1 Electrical stimulation of the heart at a rate lower than that of an existing tachycardia.

2 Electrical stimulation of the heart at a rate faster than its intrinsic rate.
